# Dual role of intraoperative ultrasound in axillary surgery: enhanced detection and surgical de-escalation in breast cancer

**DOI:** 10.1186/s12957-026-04223-8

**Published:** 2026-01-28

**Authors:** Adnan Gündoğdu, Merve Aktaş, Sangar Abdullah, Ayhan Alpar, Kübra Ertekin, Feyza Başar, Damiano Gentile, Osman Cem Yılmaz

**Affiliations:** 1 Department of General Surgery, Sancaktepe Şehit Prof. Dr. İlhan Varank Training and Research Hospital, Istanbul, Türkiye; 2 Department of General Surgery, Bayburt State Hospital, Bayburt, Türkiye; 3 Department of Radiology, Sancaktepe Şehit Prof. Dr. İlhan Varank Training and Research Hospital, Istanbul, Türkiye; 4 Department of General Surgery, Afyonkarahisar Health Sciences University Faculty of Medicine, Afyonkarahisar, Türkiye; 5 Department of Pathology, Sancaktepe Şehit Prof. Dr. İlhan Varank Training and Research Hospital, Istanbul, Türkiye; 6 Breast Unit, IRCCS Humanitas Research Hospital, Rozzano, Milan, Italy; 7 Breast Surgery, Private Clinic of Dr. Osman Cem Yılmaz, Istanbul, Türkiye

**Keywords:** Breast cancer, Intraoperative ultrasound, Sentinel lymph node biopsy, Axillary management, Surgical de-escalation

## Abstract

**Background:**

Intraoperative ultrasound (IO-USG) may optimize axillary management in breast cancer surgery. This study evaluated IO-USG’s dual role in preventing unnecessary axillary lymph node dissection (ALND) and identifying additional nodal metastases missed by sentinel lymph node biopsy (SLNB).

**Methods:**

This retrospective cohort study included 314 consecutive patients with invasive breast cancer who underwent SLNB with IO-USG evaluation between January 2019 and December 2023. IO-USG was performed after SLNB to identify suspicious non-sentinel nodes. Patients were categorized into SLNB-only, targeted axillary dissection (TAD), or ALND based on combined SLNB and IO-USG findings.

**Results:**

Of 314 patients, 113 (36%) received neoadjuvant chemotherapy and 201 (64%) underwent upfront surgery. Final surgical management comprised SLNB-only in 244 (77.7%), TAD in 46 (14.7%), and ALND in 24 (7.6%) patients. Among 89 SLNB-positive patients, 68 (76.4%) avoided ALND through IO-USG guidance. IO-USG identified additional axillary metastases in 4 of 225 SLNB-negative patients (1.8%). Molecular subtype analysis revealed no IO-USG positivity in triple-negative cases (0/25), while HR+/HER2- tumors comprised 82.6% of IO-USG-positive cases. At a median follow-up of 33.2 months, no axillary recurrences occurred.

**Conclusion:**

IO-USG may support personalized axillary management, helping avoid ALND in 76.4% of node-positive patients while identifying additional axillary metastases in 1.8% of SLNB-negative cases. As a complementary technique to standard SLNB, it appears to provide selective utility based on tumor biology.

**Trial registration:**

Not applicable.

**Supplementary Information:**

The online version contains supplementary material available at 10.1186/s12957-026-04223-8.

## Background

Axillary management in breast cancer has evolved considerably over the past decade. Sentinel lymph node biopsy (SLNB) has largely replaced axillary lymph node dissection (ALND), providing equivalent staging accuracy with markedly reduced morbidity [1]. However, false-negative rates remain a clinical concern. Wang et al. reported an 11.3% false-negative rate with SLNB alone, which decreased to 2.8% when ultrasound-suspicious nodes were also sampled [2]. In the neoadjuvant setting, Cao et al. found a pooled false-negative rate of 15% among initially node-positive patients [3]. These data highlight the ongoing need for techniques that can improve the accuracy of axillary assessment.

Preoperative axillary ultrasound has become a standard component of nodal assessment [4]. Intraoperative ultrasound (IO-USG) is now increasingly used to help surgeons identify and remove suspicious lymph nodes during breast surgery [5, 6]. Performing ultrasound in the operating room enables detection of abnormal nodes that may be missed by sentinel mapping, allows real-time confirmation of complete node removal, and helps avoid the need for a second operation [7]. Among patients who have received neoadjuvant therapy, IO-USG–guided targeted axillary dissection (TAD) has been shown to reduces false-negative rates to 4–7% when both clipped and sentinel nodes are removed in combination [8].

Recent trials have questioned the need for axillary surgery in selected patients. The SOUND [9] and INSEMA [10] trials showed that clinically node-negative patients with normal imaging can safely omit SLNB when receiving systemic therapy and radiation. Yet many patients remain ineligible for this approach—those with multifocal disease, aggressive subtypes, or planned mastectomy still require accurate staging. Identifying nodal metastases without performing full ALND remains clinically valuable. Intraoperative ultrasound could address this gap by combining sentinel node histology with real-time imaging to detect metastatic nodes that may be missed by standard mapping. This approach may reduce false-negatives and prevent reoperations, though evidence in the upfront surgery setting remains limited.

Therefore, this study evaluated IO-USG’s dual function in primary breast cancer surgery: detecting non-sentinel metastases to improve staging accuracy and enabling surgical de-escalation to avoid unnecessary ALND. Secondary objectives included identifying factors predicting IO-USG positivity and assessing long-term oncologic outcomes.

## Material and methods

### Study design

This retrospective cohort study included 314 consecutive patients with primary invasive breast cancer who underwent surgery between January 2019 and December 2023 at a high-volume breast surgery clinic. The study was reported according to STROBE (Strengthening the Reporting of Observational Studies in Epidemiology) guidelines. All patients underwent SLNB followed by IO-USG. The study was approved by the Institutional Review Board (Decision number: 2025/296) and conducted in accordance with the Declaration of Helsinki.

### Patient selection

Female patients aged 18 years or older with histologically confirmed invasive breast carcinoma (clinical stage T1-T4, N0-N3, M0) were included. Exclusion criteria were: prior ipsilateral axillary surgery, excisional lymph node biopsy for diagnosis, inflammatory breast cancer, distant metastases at diagnosis, male gender, and incomplete follow-up data.

### Preoperative assessment

All patients underwent comprehensive evaluation including bilateral mammography and/or tomosynthesis, breast and axillary ultrasound, and MRI when indicated (multifocal/multicentric disease, invasive lobular carcinoma, discordant findings). Suspicious lymph nodes were confirmed as metastatic by fine-needle aspiration cytology or core needle biopsy before treatment initiation. Treatment decisions were determined by a multidisciplinary tumor board consisting of breast surgeons, medical oncologists, radiation oncologists, radiologists, and pathologists.

### Treatment allocation

Neoadjuvant chemotherapy was administered to 113 patients (36.0%) following contemporary NCCN and AGO recommendations [10, 11]. Among these, 86 had clinical node-positive disease, while 27 had node-negative disease but received NAC for tumor downstaging (cT3-T4), aggressive biology (HER2+/TNBC), or to enable breast conservation.Of 99 patients with clinical node-positive disease at diagnosis, 86 (86.9%) received NAC while 13 (13.1%) underwent upfront surgery due to favorable HR+/HER2- biology (*n* = 12) or patient preference (*n* = 1).

### Neoadjuvant monitoring

Lymph node markers were placed in 43/86 (50%) node-positive NAC patients when technically feasible. Response assessment demonstrated that 63 of 86 (73.3%) patients achieved complete clinical response (ycN0), while 23 (26.7%) had persistent disease (ycN+).

### Surgical technique

Sentinel lymph node biopsy was performed using 1% isosulfan blue dye injected periareolarly. Isosulfan blue was used as the institutional standard; indocyanine green (ICG) was not available during the study period. Blue-stained sentinel nodes were identified through a standard axillary incision. All intraoperative axillary ultrasound (IO-USG) examinations were performed by a single breast surgeon with more than 10 years of experience in intraoperative ultrasound. A high-frequency linear probe (L4–15, 4–15 MHz; Esaote MyLab Sigma, Italy) was used with standardized depth and gain settings optimized to delineate cortical–hilum interfaces.

IO-USG was conducted in two phases: first, transcutaneous scanning before SLNB to map axillary anatomy and identify grossly suspicious nodes; second, direct scanning on exposed axillary tissue after SLNB to confirm complete sentinel retrieval and detect suspicious non-sentinel nodes. Systematic sweeps of axillary levels I–II were performed, with selective evaluation of level III only when cortical irregularity or nodal asymmetry in lower levels suggested higher-level disease .

Suspicious findings were predefined and included cortical thickness > 3 mm, eccentric cortical hypertrophy, partial or complete effacement of the fatty hilum, and non-hilar peripheral vascularity [8, 12]. When present, suspicious nodes were excised under real-time ultrasound guidance. Frozen section analysis was performed on all sentinel nodes. The surgeon performing IO-USG was blinded to SLNB results, as frozen-section evaluation was completed later in the procedure, whereas IO-USG assessment occurred immediately after node exposure.

### Surgical decision algorithm

**Fig. 1:**
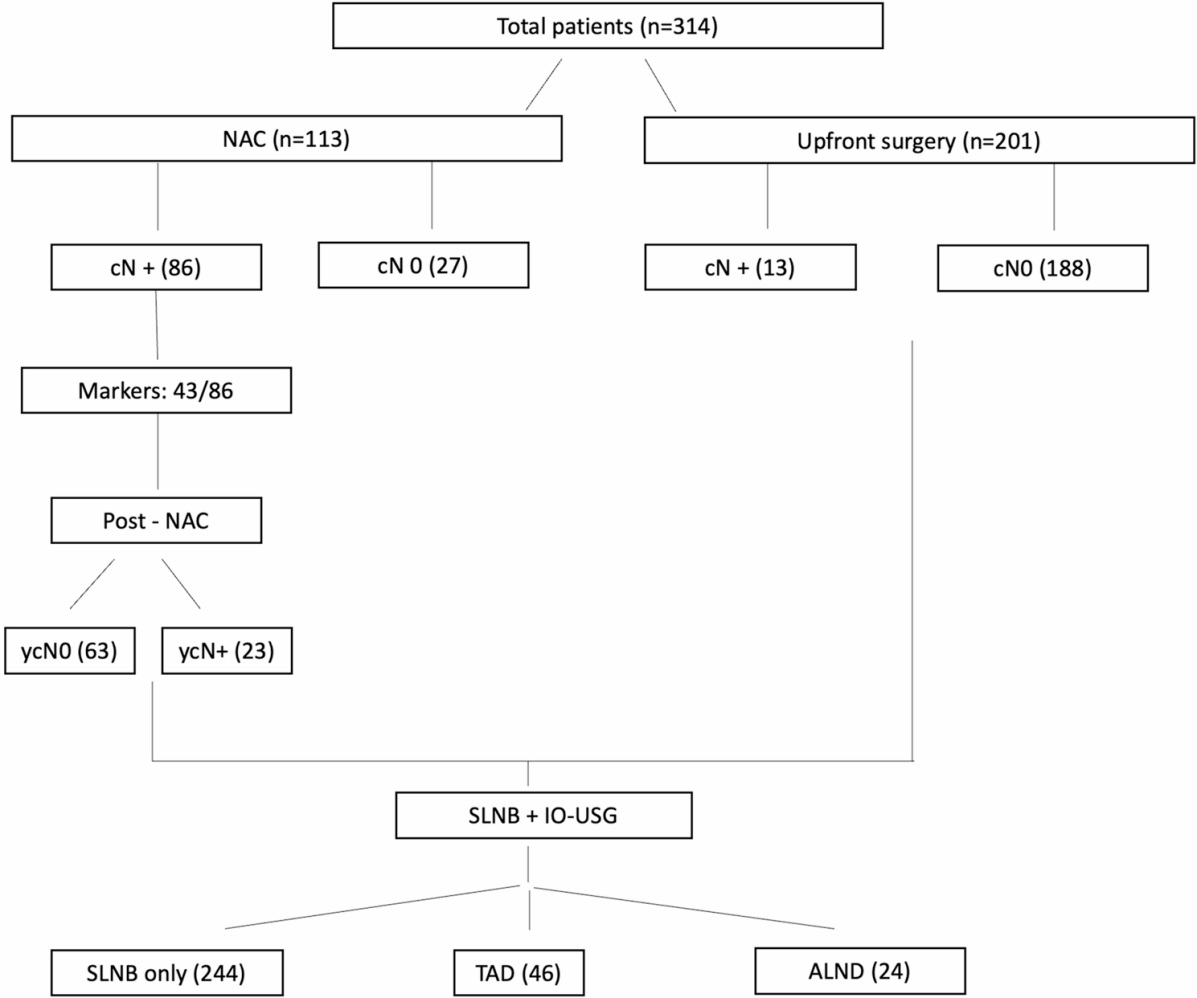
Patient flow diagram showing distribution across surgical management groups based on SLNB and intraoperative ultrasound findings

Final axillary management was determined intraoperatively based on the combined assessment of SLNB and IO-USG. The patient flow and surgical decision pathway are illustrated in Fig. 1. Patients were classified into three groups:

### Group 1 – SLNB-only (*n* = 244)

This group included patients with negative SLNB and negative IO-USG (*n* = 210), as well as patients with 1–2 SLNs showing macrometastasis on frozen section but with negative IO-USG who met Z0011 criteria and therefore did not require further axillary surgery (*n* = 34).

### Group 2 – Targeted axillary dissection (TAD) (*n* = 46)

Patients in whom IO-USG identified a discrete suspicious non-sentinel node underwent TAD under real-time ultrasound guidance. These patients generally had limited SLN tumor burden on frozen Sects. (1–2 macrometastatic SLNs), and IO-USG-guided removal focused exclusively on the sonographically suspicious node(s).

### Group 3 – Axillary lymph node dissection (ALND) (*n* = 24)

Completion ALND was performed for patients with ≥ 3 macrometastatic SLNs on frozen section, clinically N2–N3 disease, or other intraoperatively assessed high-burden features. ALND was also undertaken in selected cases with occult primary breast cancer or equivocal frozen-section findings according to institutional protocol. Notably, ALND in this cohort was driven by SLNB/frozen findings and clinical criteria rather than IO-USG, as IO-USG did not demonstrate suspicious nodes in this group.

### Statistical analysis

All statistical analyses were performed using R software (version 4.2.3; R Foundation for Statistical Computing, Vienna, Austria) with the stats, survival, and ggplot2 packages. Continuous variables were expressed as mean ± standard deviation (SD) or median (interquartile range, IQR) according to data distribution, assessed by the Shapiro–Wilk test. Between-group comparisons were performed using the Student’s t-test or Mann–Whitney U test as appropriate. Categorical variables were presented as numbers (percentages) and compared using the chi-square or Fisher’s exact test.

Univariable logistic regression analyses were used to identify predictors of non-sentinel lymph node metastasis. Variables with *p* < 0.10 in univariable analysis were included in the multivariable logistic regression model. Odds ratios (ORs) with 95% confidence intervals (CIs) were calculated. All tests were two-sided, and *p* < 0.05 was considered statistically significant. No correction for multiple testing was applied, as analyses were exploratory.

Survival outcomes were estimated using the Kaplan–Meier method, and survival curves were compared using the log-rank test. Median follow-up duration was calculated using the reverse Kaplan–Meier method. Overall survival (OS), distant relapse-free survival (DRFS), and invasive disease-free survival (IDFS) were calculated from the date of surgery to the date of the event or last follow-up.

## Results

A total of 314 patients underwent surgery with SLNB between January 2019 and December 2023. The mean age was 51.6 ± 11.8 years, and most tumors were invasive ductal carcinoma (78.7%) with HR+/HER2– subtype predominance (74.2%). Clinical T1–T2 disease was present in 93.0%, and 36.0% received NAC. Baseline characteristics are presented in Table 1. Comparison between NAC and upfront surgery groups is shown in Table 2.

**Table 1:** Baseline and pathological characteristics of the study cohort (*n* = 314)

Variable	Overall (*n* = 314)	SLNB (*n* = 244)	ALND (*n* = 24)	TAD (*n* = 46)
Age (years)	51.6 ± 11.8	51.9 ± 12.0	52.5 ± 11.0	49.8 ± 11.2
Histology				
Ductal carcinoma	247 (78.7)	197 (80.7)	16 (66.7)	34 (73.9)
Lobular carcinoma	33 (10.5)	21 (8.6)	6 (25)	6 (13)
Mixed/Other	34 (10.8)	26 (10.7)	2 (8.3)	6 (13)
Molecular subtype				
HR+/HER2–	233 (74.2)	172 (70.5)	23 (95.8)	38 (82.6)
HR+/HER2+	37 (11.8)	32 (13.1)	0 (0%)	5 (10.9)
HR–/HER2+	19 (6.1)	15 (6.1)	1 (4.2)	3 (6.5)
Triple-negative	25 (8)	25 (10.2)	0 (0)	0 (0)
Ki-67 ≥ 10%, n (%)	242 (77.1)	190 (77.9)	18 (75)	34 (73.9)
Clinical Tumor features				
Clinical T1	137 (43.6)	116 (47.5)	6 (25)	15 (32.6)
Clinical T2	155 (49.4)	116 (47.5)	13 (54.2)	26 (56.5)
Clinical T3–T4	22 (7.0)	12 (5)	5 (20.8)	5 (10.9)
Clinical N0	215 (68.5)	185 (75.8)	10 (41.7)	20 (43.5)
Clinical N1–N3	99 (31.5)	59 (24.2)	14 (58.3)	26 (56.5)
Multifocal/multicentric	115 (36.6)	84 (34.4)	8 (33.3)	23 (50)
Magseed placement	20 (18.7%)	15 (19.7%)	1 (9.1%)	4 (20%)
Clip placement	23 (21.5%)	21 (27.6%)	1 (9.1%)	1 (5%)
Pathologic T stage				
Pathologic T0	43 (13.7)	40 (16.4)	1 (4.2)	2 (4.3)
Pathologic T1	157 (50)	124 (50.8)	11 (45.8)	22 (47.8)
Pathologic T2–T3	114 (36.3)	80 (32.7)	12 (50.0)	22 (47.9)
Pathologic N stage ≥ N1	91 (29.0)	38 (15.4)	21 (87.5)	32 (72.7)
Histological grade				
Low	25 (10.3)	23 (12.4)	0 (0)	2 (5.4)
Intermediate	153 (63.2)	115 (61.8)	12 (63.2)	26 (70.3)
High	64 (26.4)	48 (25.8)	7 (36.8)	9 (24.3)
Total sentinel nodes (mean ± SD)	2.9 ± 1.4	2.9 ± 1.4	3.1 ± 1.8	2.9 ± 1.3
Positive non-sentinel nodes (mean ± SD)	0.5 ± 0.9	0.2 ± 0.5	2 ± 1.8	1.1 ± 1
USG-guided targeted suspicious nodes	0.5 ± 1.4	0 ± 0	0 ± 0	3.4 ± 1.9
USG-guided positive nodes	0.1 ± 0.5	0 ± 0	0 ± 0	0.7 ± 1
Macrometastasis	0.2 ± 0.4	0.1 ± 0.3	0.9 ± 0.3	0.6 ± 0.5
Micrometastasis	0.1 ± 0.3	0.1 ± 0.2	0.2 ± 0.4	0.2 ± 0.4
Lymphovascular invasion	80 (28.1)	47 (21.5)	15 (68.2)	18 (40.9)
Perinodal invasion	31 (9.9)	6 (2.5)	15 (62.5)	10 (21.7)
Treatment				
Neoadjuvant chemotherapy	113 (36)	81 (33.2)	11 (45.8)	21 (45.7)
Adjuvant radiotherapy	242 (77.1)	181 (74.2)	21 (87.5)	40 (87)
Adjuvant hormone therapy	268 (85.4)	203 (83.2)	23 (95.8)	42 (91.3)
Preoperative USG findings				
Suspicious	59 (18.8)	20 (8.2)	16 (66.7)	23 (50)
Negative	255 (81.2)	224 (91.8)	8 (33.3)	23 (50)
Axillary assessment				
SLNB positive	89 (28.3)	37 (15.2)	21 (87.5)	31 (67.4)
Intraoperative USG positive	46 (14.6)	0 (0)	0 (0)	46 (100)
No recurrence	300 (95.5)	236 (96.7)	20 (83.3)	44 (95.7)
Death	5 (1.6)	3 (1.2)	2 (8.3)	0 (0)

Data are presented as mean ± standard deviation or number (percentage)

Continuous variables were analyzed with the Shapiro-Wilk test; categorical variables with the χ² or Fisher’s exact test, as appropriate

Among 25 triple-negative patients, 15 received neoadjuvant chemotherapy with one patient having residual micrometastatic disease. The remaining 10 underwent upfront surgery, all being clinically node-negative

*Abbreviations: SLNB* Sentinel lymph node biopsy, *ALND* Axillary lymph node dissection, *TAD* Targeted axillary dissection, *USG* Ultrasound, *HR* Hormone receptor, *HER2* Human epidermal growth factor receptor 2

**Table 2:** Comparison of clinicopathological features between neoadjuvant chemotherapy and upfront surgery groups

	Overall (*n* = 314)	NAC (*n* = 113)	Upfront (*n* = 201)	*p* - value
Age (years)	50 (43-59.8)	46 (40–54)	52 (45–62)	< 0.001ᵞ
Clinical T stage				< 0.001ᶿ
T1	137 (43.6%)	22 (19.5%)	115 (57.2%)	
T2	155 (49.4%)	69 (61.1%)	86 (42.8%)	
T3	18 (5.7%)	18 (15.9%)	0	
T4	4 (1.3%)	4 (3.5%)	0	
Clinical N stage				< 0.001ᶿ
N0	215 (68.5%)	27 (23.9%)	188 (93.5%)	
N1	72 (22.9%)	59 (52.2%)	13 (6.5%)	
N2	19 (6.1%)	19 (16.8%)	0	
N3	8 (2.5%)	8 (7.1%)	0	
Histological subtype				0.001ᶿ
Ductal carcinoma	247 (78.7%)	100 (88.5%)	147 (73.1%)	
Lobular carcinoma	33 (10.5%)	7 (6.2%)	26 (12.9%)	
Mix	19 (6.1%)	6 (5.3%)	13 (6.5%)	
Other	15 (4.8%)	0	15 (7.5%)	
Molecular subtype				< 0.001ᶿ
HR+ / HER2-	233 (74.2%)	58 (51.3%)	175 (87.1%)	
HR+ / HER2+	37 (11.8%)	23 (20.4%)	14 (7%)	
HR- / HER2+	19 (6.1%)	17 (15%)	2 (1%)	
TN	25 (8%)	15 (13.3%)	10 (5%)	
Preoperative suspicious ultrasound findings	59 (18.8%)	33 (29.2%)	26 (12.9%)	< 0.001ᵸ
Intraoperative ultrasound positive	46 (14.6%)	21 (18.6%)	25 (12.4%)	0.139ᵸ
Surgical procedure				0.157ᵸ
SLNB	244 (77.7%)	81 (71.7%)	163 (81.1%)	
ALND	24 (7.6%)	11 (9.7%)	13 (6.5%)	
TAD	46 (14.6%)	21 (18.6%)	25 (12.4%)	

*Abbreviations: NAC* Neoadjuvant chemotherapy, *HR* Hormone receptor, *HER2* Human epidermal growth factor receptor 2, *TN* Triple negative, *SLNB* Sentinel lymph node biopsy, *ALND* Axillary lymph node dissection, *TAD* Targeted axillary dissection

ᵞ Mann-Whitney U test; ᶿ Fisher’s exact test; ᵸ Pearson’s Chi-square test

Final axillary management consisted of SLNB-only in 244 patients (77.7%), TAD in 46 (14.7%), and ALND in 24 (7.6%). The patient flow and surgical decision pathway are illustrated in Fig. 1. Overall, nodal metastasis (≥ pN1) was detected in 92 patients (29.3%). Intraoperative ultrasound identified suspicious non-sentinel nodes in 46 patients (14.7%), all of whom underwent TAD. Among 89 SLNB-positive patients, 68 (76.4%) avoided completion ALND: thirty-seven had negative IO-USG and required no further surgery, while thirty-one underwent IO-USG–guided removal of the suspicious node(s). Twenty-one patients (23.6%) proceeded to ALND because of high-burden findings on SLNB or clinical factors.

Among 225 SLNB-negative patients, 15 (6.7%) had suspicious IO-USG findings requiring TAD, with metastases confirmed in 3 (20%). While 12 cases proved false-positive, 9 of these (75%) had only 1–2 additional nodes removed, minimizing surgical extent. Three additional SLNB-negative patients underwent ALND despite negative IO-USG: one for occult primary (no metastasis found) and two for false-positive frozen sections (one harboring metastatic disease). Overall, IO-US identified additional metastases in 1.8% (4/225) of SLNB-negative patients.

Detailed diagnostic performance of IO-US, including cross-tabulation of preoperative US, intraoperative US, and final pathology, is presented in Tables 3, 4, 5, 6, 7. All marked lymph nodes (23 clips, 20 Magseeds) were successfully retrieved under IO-US guidance.

**Table 3:** Positive predictive value of IO-US in NAC and upfront surgery subgroups

Group	IO-US Positive (*n*)	True Positive	False Positive	PPV (95% CI)
NAC Group	21	15	6	71.4% (47.8–88.7)
Upfront Surgery	25	5	20	20% (6.8–40.7)

Fisher’s exact test P-value = < 0.001 (significant difference between groups)

True-positive and false-positive values were available only in IO-US–positive patients who underwent targeted nodal excision. IO-US–negative patients did not undergo ALND; therefore, false-negative and true-negative rates cannot be determined

*PPV* Positive predictive value, *NAC* Neoadjuvant chemotherapy, *IO-US* Intraoperative ultrasonography

**Table 4:** Cross-tabulation of preoperative USG and intraoperative USG

Preoperative USG	IO-US Positive	IO-US Negative	Total
Suspicious	23	36	59
Negative	23	232	255
**Total**	**46**	**268**	**314**

Preoperative USG ‘suspicious’ refers to cortical thickness > 3 mm, eccentric cortical hypertrophy, loss of fatty hilum, or non-hilar peripheral vascularity

*IO-US* Intraoperative ultrasonography

**Table 5:** Cross-tabulation: preoperative USG vs final pathology

Preoperative USG	Final Pathology Positive	Final Pathology Unknown	Total
Suspicious	45	14	59
Negative	47	208	255
**Total**	**92**	**222**	**314**

Final pathology “unknown” indicates patients with negative sentinel lymph node biopsy who did not undergo ALND; therefore, non-sentinel lymph nodes were not pathologically assessed

**Table 6:** Cross-tabulation of intraoperative USG and final pathology

Intraoperative USG	Final Pathology Negative	Final Pathology Positive	Final Pathology Unknown	Total
Positive	26	20	0	46
Negative	0	0	268	268
**Total**	**26**	**20**	**268**	**314**

Pathologic confirmation was available only for IO-US–positive patients who underwent ultrasound-guided excision of the suspicious node(s)

IO-US–negative patients did not undergo completion ALND; therefore, the presence or absence of additional nodal metastasis could not be verified, and true-negative or false-negative rates cannot be determined

**Table 7:** SLNB-negative & IO-US-positive subgroup analysis

Outcome	n
Targeted Axillary Pathology Positive	3
Targeted Axillary Pathology Negative	12

All SLNB-negative, IO-US–positive patients underwent limited IO-US–guided excision only; none proceeded to completion ALND

IO-USG positivity was strongly associated with clinical nodal stage (*p* < 0.001), while no IO-USG-positive findings occurred in triple-negative tumors. Other clinicopathologic correlations are shown in Table 8. In multivariable analysis, lobular/mixed histology and clinical N2–N3 status independently predicted non-sentinel node metastasis (Table 9).

**Table 8:** Clinicopathological characteristics according to intraoperative ultrasound findings

Variable	Overall (*n* = 314)	IO-USG Positive (*n* = 46)	IO-USG Negative (*n* = 268)	*p* value
Histological subtype				0.057ᶿ
Ductal carcinoma	247 (78.7)	34 (73.9)	213 (79.5)	
Lobular carcinoma	33 (10.5)	6 (13)	27 (10.1)	
Mixed	19 (6.1)	6 (13)	13 (4.9)	
Other	15 (4.8)	0 (0)	15 (5.6)	
Molecular subtype				0.122ᶿ
HR+/HER2–	233 (74.2)	38 (82.6)	195 (72.8)	
HR+/HER2+	37 (11.8)	5 (10.9)	32 (11.9)	
HR–/HER2+	19 (6.1)	3 (6.5)	16 (6)	
Triple-negative	25 (8)	0 (0)	25 (9.3)	
Ki-67 index, ≥ 10%	242 (77.1)	34 (73.9)	208 (77.6)	0.581ᵸ
Tumor features				
Clinical T stage				0.175ᶿ
T1	137 (43.6)	15 (32.6)	122 (45.5)	
T2	155 (49.4)	26 (56.5)	129 (48.1)	
T3–T4	22 (7.0)	5 (10.9)	17 (6.4)	
Clinical N stage				< 0.001ᶿ
N0	215 (68.5)	20 (43.5)	195 (72.8)	
N1	72 (22.9)	16 (34.8)	56 (20.9)	
N2–N3	27 (8.6)	10 (21.7)	17 (6.4)	

Data are presented as number (percentage)

Continuous variables were analyzed using the Shapiro–Wilk test; categorical variables were compared with the χ² or Fisher’s exact test, as appropriate

*HR* Hormone receptor, *HER2* Human epidermal growth factor receptor 2, *USG* Ultrasound

ᶿ Fisher’s exact test; ᵸ Pearson’s Chi-square test

**Table 9:** Univariable and multivariable logistic regression analysis of preoperative predictors for Non-Sentinel lymph node metastasis in patients

Variable	Univariable Analysis			Multivariable Analysis		
	OR	95% CI	*p*	OR	95% CI	*p* value
Age (years)	0.975	0.941–1.008	0.137	0.967	0.926–1.008	0.116
Histological subtype			< 0.001			0.011
Ductal carcinoma	Reference	—	—	Reference	—	—
Lobular carcinoma	3.643	1.308–9.328	—	5.465	1.472–21.140	—
Mixed	6.244	1.991–18.063	—	5.571	1.355–22.941	—
Molecular subtype			0.021			0.012
HR+/HER2–	Reference	—	—	Reference	—	—
HR+/HER2+	0.203	0.011–1.001	—	0.134	0.007–0.793	—
HR–/HER2+	0.407	0.022–2.092	—	0.247	0.012–1.767	—
Ki-67 index (≥ 10%)	0.800	0.352–1.992	0.614	0.654	0.205–2.128	0.472
Clinical T stage			< 0.001			0.131
T1	Reference	—	—	Reference	—	—
T2	3.469	1.340–10.746	—	3.066	1.022–10.692	—
T3	16.800	4.651–65.838	—	4.184	0.776–24.060	—
Clinical N stage			< 0.001			0.001
N0	Reference	—	—	Reference	—	—
N1	4.128	1.635–10.686	—	8.731	2.194–38.113	—
N2	13.352	4.163–42.534	—	27.725	4.347–204.629	—
N3	13.733	2.516–65.839	—	22.025	2.272–225.072	—
Clinical multifocal/multicentric	1.172	0.530–2.507	0.688	0.666	0.227–1.855	0.440
Neoadjuvant chemotherapy	3.491	1.621–7.872	0.001	0.829	0.182–3.508	0.802
Number of positive sentinel nodes	4.154	2.732–6.665	< 0.001	0.659	0.252–1.601	0.360
Total number of sentinel nodes	1.241	0.975–1.556	0.078	1.177	0.701–1.91	0.526

Data are expressed as odds ratio (OR) with 95% confidence intervals (CI)

Univariable and multivariable logistic regression analyses were performed to determine predictors of non-sentinel lymph node positivity

Variables with *p* < 0.10 in univariable analysis were included in the multivariable model

Categories with insufficient data were excluded from regression analysis

After a median follow-up of 33.2 months, no axillary recurrences were observed in any surgical group. Five deaths (1.6%) occurred during follow-up. The absence of axillary failure supports the oncologic safety of IO-USG–guided selective axillary surgery.

## Discussion

In this single-center experience, IO-USG demonstrated a dual role in personalizing axillary management—enabling surgical de-escalation in 76.4% of node-positive patients while detecting metastatic disease in 1.8% of sentinel node-negative cases that would have been missed by SLNB alone. Although no axillary recurrences were observed at a median follow-up of 33.2 months, this finding should be interpreted as reassuring rather than definitive evidence of long-term oncologic safety. IO-USG complements contemporary axillary-sparing techniques, including TAD, by providing real-time, anatomy-based intraoperative assessment applicable in both neoadjuvant and upfront settings [8, 13].

The key advantage of IO-USG lies in its iterative use throughout surgery. Unlike static imaging, IO-USG provides real-time assessment at multiple stages—after SLNB, following frozen section, and during TAD. This approach extends the principles of TAD beyond the post-neoadjuvant context, enabling their application across all surgical settings [8, 14, 15]. IO-USG-guided management prevented ALND in 76.4% of node-positive patients. As in Z0011 and SINODAR-ONE trials where ALND was safely omitted [16, 17], 37 patients with positive SLNB but negative IO-USG avoided further surgery, while 31 underwent limited TAD. Importantly, IO-USG identified occult metastases in 3 of 225 SLNB-negative patients. In one case, false-positive frozen section led to ALND based on IO-USG findings, revealing a single positive node, demonstrating the complexity of real-time surgical decision-making.

Importantly, IO-USG performed effectively regardless of lymph node marker availability. In this cohort, only 50% of patients receiving NAC had marker placement due to resource limitations. Nevertheless, IO-USG successfully identified residual disease in both marked and unmarked patients, suggesting that it may perform as a practical alternative or complementary tool in resource-limited environments where clip or seed technology is not routinely available.

This study’s findings are consistent with ongoing de-escalation trials. While Z0011 and AMAROS demonstrated that ALND can be safely omitted in selected patients with limited nodal involvement [16, 18], and SOUND and INSEMA questioned the necessity of SLNB in low-risk populations [9, 19], a significant proportion of patients remain ineligible for these strategies. IO-USG has demonstrated strong potential as a practical intraoperative tool for selected patient groups, particularly where advanced localization systems are unavailable.

The diagnostic performance of IO-US varied substantially between treatment settings. In the NAC cohort, the positive predictive value reached 71.4%, whereas it was only 20% in upfront surgery patients (*p* < 0.001). This difference likely reflects the higher pretest probability of residual nodal disease in patients with incomplete response after NAC, in whom suspicious nodes are more likely to harbor metastasis. In contrast, treatment-naïve patients often present with low-volume axillary disease, reducing the specificity of IO-US and increasing the rate of false-positive findings. These observations are consistent with prospective multicenter trials such as ACOSOG Z1071, SENTINA, and SN-FNAC, which reported false-negative rates of 12–14% for SLNB after NAC in initially node-positive patients, highlighting the need for additional intraoperative safeguards in this setting [20–22]. Accordingly, IO-US may serve as a complementary tool after NAC to identify residual disease not detected by SLNB. Conversely, in upfront surgery patients, the low PPV suggests that IO-US should be integrated cautiously into surgical decision-making, serving to support rather than mandate additional axillary resection.

Lobular histology emerged as the strongest independent predictor of non-sentinel metastasis (OR 5.5, 95% CI 1.5–21.1), consistent with Garcia-Tejedor et al.‘s findings [23]. Clinical N2-N3 disease showed elevated odds ratios (OR 22.0-27.7, *p* = 0.001), providing clear risk stratification. These findings suggest IO-USG yields greatest benefit in patients with lobular/mixed histology and advanced clinical nodal involvement. Future multicenter prospective trials with standardized IO-USG protocols would better define its diagnostic accuracy and establish selection criteria for its optimal use.

Molecular subtype analysis revealed distinct IO-USG utility. None of 25 TNBC patients showed IO-USG positivity, with all initially node-positive cases achieving complete response after NAC, consistent with contemporary pCR rates exceeding 60% [24, 25]. Similarly, HER2-positive disease showed low odds of non-sentinel metastasis (OR 0.134, *p* = 0.012). In contrast, HR+/HER2- tumors comprised 82.6% of IO-USG-positive cases, reflecting their limited chemosensitivity. These findings support a biology-driven application of IO-USG—routine use in HR+/HER2 − disease and selective use in TNBC or HER2-positive subtypes as a safety measure for detecting incomplete response.

Micrometastasis rates were similarly low (0.1–0.2) across all groups, while macrometastasis increased with extent of surgery (SLNB 0.2, TAD 0.6, ALND 0.9), confirming IO-USG’s selective detection of clinically significant disease. Our zero recurrence rate suggests adjuvant therapy effectively controlled any missed microscopic disease, aligning with Z0011 and AMAROS trials where radiotherapy provided excellent regional control without complete surgical clearance [18, 26].

Avoiding ALND in 76.4% of node-positive patients prevented significant morbidity, including lymphedema (15–30%), chronic pain, and shoulder dysfunction [27, 28]. Additionally, IO-USG’s independence from radioactive tracers or specialized equipment makes it globally accessible, particularly in resource-limited settings.

This study has several limitations. The retrospective single-center design may introduce selection bias, and single-surgeon experience limits generalizability. Furthermore, the learning curve and interobserver variability for IO-USG remain undefined. The true false-negative rate cannot be determined as IO-USG-negative patients did not undergo routine ALND; however, no axillary recurrences occurred during follow-up. The 50% lymph node marker rate in NAC patients was suboptimal due to resource constraints. Additionally, retrospective review suggests some patients who underwent ALND for limited disease might have been managed with TAD, particularly those with low tumor burden. The high false-positive rate of IO-US in upfront surgery patients (80%) requires validation in larger prospective studies before widespread adoption in this population.

Future multicenter prospective trials comparing SLNB alone versus SLNB + IO-USG with standardized protocols are needed. Economic analysis would strengthen the value proposition, as avoiding ALND in 76.4% of node-positive patients likely reduces costs through decreased morbidity and shorter hospital stays.

## Conclusion

Intraoperative ultrasound may support personalized axillary management in breast cancer surgery. In this cohort, it helped avoid ALND in 76.4% of node-positive patients while identifying additional axillary metastases in 1.8% of SLNB-negative cases. IO-USG appears to offer selective utility based on tumor biology, with greater potential benefit in HR+/HER2- and lobular carcinomas. As a complementary technique to standard sentinel node mapping, IO-USG warrants further prospective evaluation to define its role in optimizing axillary staging.

## Supplementary Information

Supplementary Material 1. Supplementary Figure 1. Intraoperative workflow of sentinel lymph node biopsy and axillary ultrasound assessment. (A) Periareolar injection of 1% isosulfan blue dye. (B) Identification of blue-stained sentinel lymph nodes. (C) Intraoperative ultrasound using a high-frequency linear probe. (D) Excised sentinel lymph nodes.

Supplementary Material 2. Supplementary Figure 2. Kaplan–Meier survival analysis of 314 patients. (A) Distant relapse-free survival (DRFS). (B) Invasive disease-free survival (IDFS). (C) Overall survival (OS).

Supplementary Material 3.

Supplementary Material 4.

## Data Availability

The datasets used and/or analysed during the current study are available from the corresponding author on reasonable request.

## Abbreviations

AGO: Arbeitsgemeinschaft Gynäkologische Onkologie
ALND: Axillary Lymph Node Dissection
CI: Confidence Interval
DRFS: Distant Relapse-Free Survival
HER2: Human Epidermal Growth Factor Receptor 2
HR: Hormone Receptor
IDFS: Invasive Disease-Free Survival
IO-USG: Intraoperative Ultrasound
IQR: Interquartile Range
MRI: Magnetic Resonance Imaging
NAC: Neoadjuvant Chemotherapy
NCCN: National Comprehensive Cancer Network
OR: Odds Ratio
OS: Overall Survival
pCR: Pathological Complete Response
SD: Standard Deviation
SLNB: Sentinel Lymph Node Biopsy
STROBE: Strengthening the Reporting of Observational Studies in Epidemiology
TAD: Targeted Axillary Dissection
TNBC: Triple-Negative Breast Cancer
