# Supplementary figures and images for: Dual role of intraoperative ultrasound in axillary surgery: enhanced detection and surgical de-escalation in breast cancer
Source: World J Surg Oncol. 2026 Jan 28;24:95. doi: 10.1186/s12957-026-04223-8 (PMC12924507; doi:10.1186/s12957-026-04223-8)

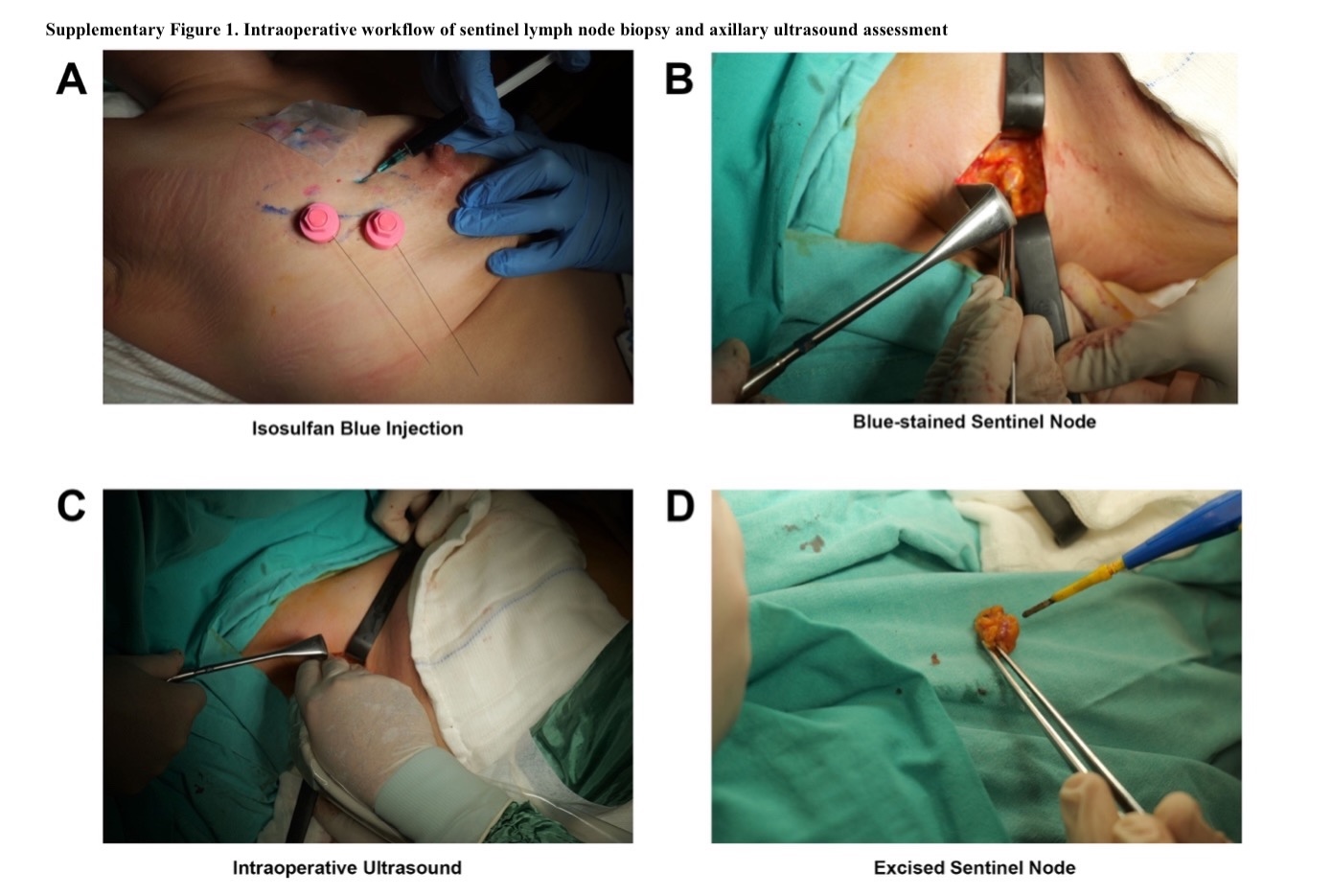

Supplement: Supplementary file 1 — Supplementary Material 1. Supplementary Figure 1. Intraoperative workflow of sentinel lymph node biopsy and axillary ultrasound assessment. (A) Periareolar injection of 1% isosulfan blue dye. (B) Identification of blue-stained sentinel lymph nodes. (C) Intraoperative ultrasound using a high-frequency linear probe. (D) Excised sentinel lymph nodes. [file 12957_2026_4223_MOESM1_ESM.jpg]

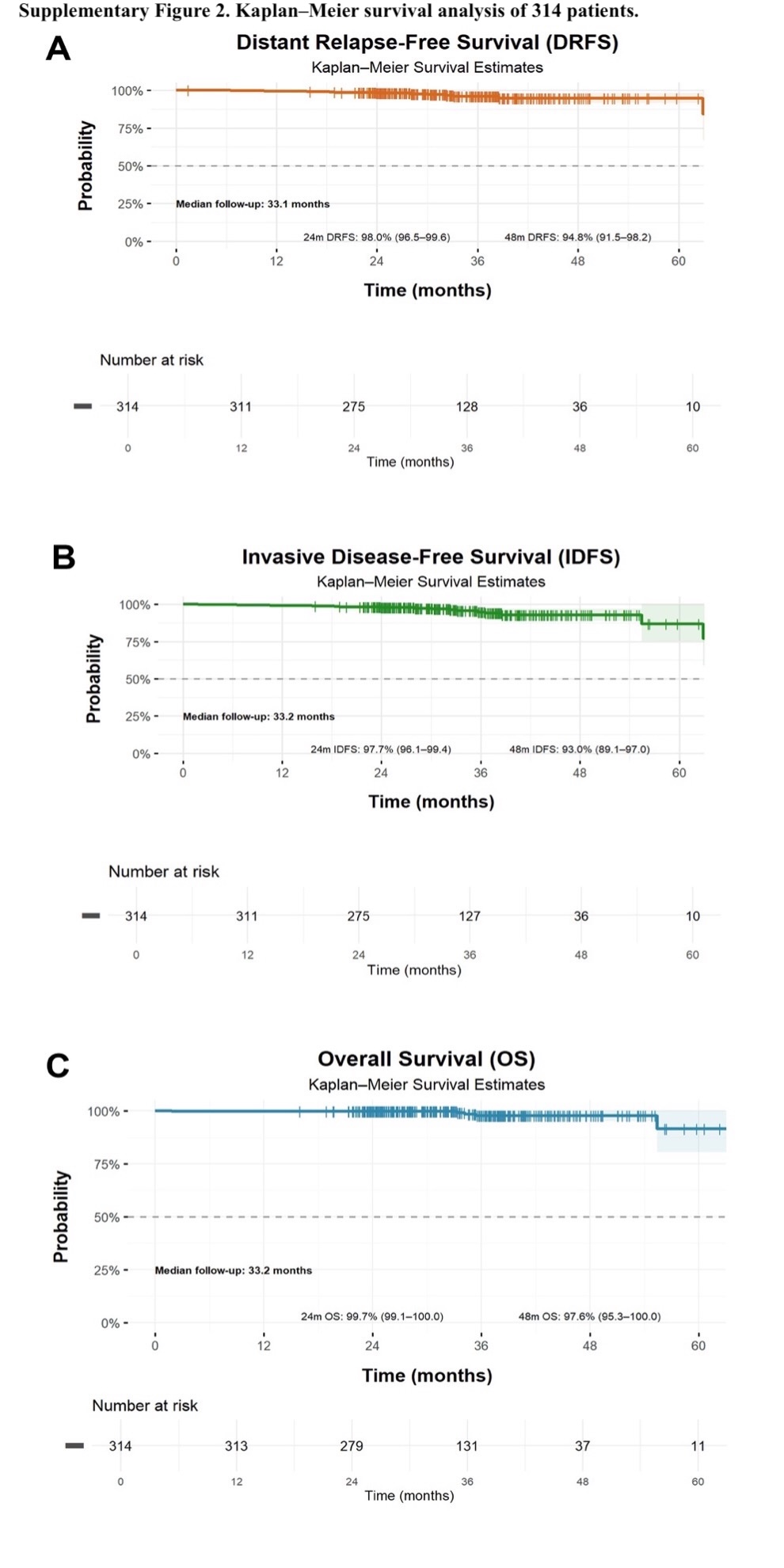

Supplement: Supplementary file 2 — Supplementary Material 2. Supplementary Figure 2. Kaplan–Meier survival analysis of 314 patients. (A) Distant relapse-free survival (DRFS). (B) Invasive disease-free survival (IDFS). (C) Overall survival (OS). [file 12957_2026_4223_MOESM2_ESM.jpg]
