# Supplementary material for: Dual role of intraoperative ultrasound in axillary surgery: enhanced detection and surgical de-escalation in breast cancer
Source: World J Surg Oncol. 2026 Jan 28;24:95. doi: 10.1186/s12957-026-04223-8 (PMC12924507; doi:10.1186/s12957-026-04223-8)
Supplement: Supplementary file 3 — Supplementary Material 3. [file 12957_2026_4223_MOESM3_ESM.pdf]

T.C.
İSTANBUL VALİLİĞİ
İl Sağlık Müdürlüğü
Şehit Prof. Dr. İlhan Varank Eğitim ve Araştırma Hastanesi

Sayı : E-46059653-050.04-286650422 29.08.2025
Konu : Uzm. Dr. Adnan GÜNDOĞDU Bilimsel Araştırmalar Etik Kurul Toplantısı Hk

Sayın Uzm. Dr. Adnan GÜNDOĞDU

Hastanemiz Sancaktepe Şehit Prof. Dr. İlhan Varank Eğitim ve Araştırma Hastanesinde 27.08.2025 tarihinde yapılan Bilimsel Araştırmalar Etik Kurul toplantısında Ek 1 de yer alan bir adet dosyanız "İntraoperatif Ultrasonun Çift Yönlü Kullanımı: Aksiller Cerrahide Tanı ve Deskalasyon" isimli retrospektif bilimsel çalışmanızın gerçekleştirilmesinde etik açıdan bir sakınca olmadığına toplantıya katılan etik kurul üye tam sayısının salt çoğunluğu ile karar verilmiştir.

2025/296 sayı numaralı Kurul Karar Formu Ek-2 de yer almaktadır.

Gereğini rica ederim.

Doç. Dr. ORHUN SİNANOĞLU
Etik Kurul Başkanı

Ek:
1 - UZM. DR.ADNAN GÜNDOĞDU BİLİMSEL ARAŞTIRMALAR ETİK KURUL DOSYASI
2 - UZM. DR. ADNAN GÜNDOĞDU BİLİMSEL ARAŞTIRMALAR ETİK KURUL KARAR FORMU

Emek Mah.Namık Kemal Cad.No:54 Sancaktepe İSTANBUL 34000
Telefon No: 02166063300
e-Posta: İnternet Adresi: https://www.saglik.gov.tr/
Kep Adresi:
Bilgi için: Fatma ATA
Asistan
Telefon No: 21660633001311
