# Supplementary material for: Dual role of intraoperative ultrasound in axillary surgery: enhanced detection and surgical de-escalation in breast cancer
Source: World J Surg Oncol. 2026 Jan 28;24:95. doi: 10.1186/s12957-026-04223-8 (PMC12924507; doi:10.1186/s12957-026-04223-8)
Supplement: Supplementary file 4 — Supplementary Material 4. [file 12957_2026_4223_MOESM4_ESM.doc]

STROBE Statement—Checklist of items that should be included in reports of ***cohort studies***

Manuscript Title: "Dual Role of Intraoperative Ultrasound in Axillary Surgery: Enhanced Detection and Surgical De-escalation in Breast Cancer"

|  | Item No | Recommendation |
| --- | --- | --- |
| **Title and abstract** | 1 | (a) Indicate the study’s design with a commonly used term in the title or the abstract  **✓ YES - Abstract mentions "retrospective cohort study" (Methods section)** |
|  |  | (b) Provide in the abstract an informative and balanced summary of what was done and what was found  ✓ YES - Abstract contains structured Background, Methods, Results, Conclusions |
| Introduction | | |
| Background/rationale | 2 | Explain the scientific background and rationale for the investigation being reported  ✓ YES - Introduction paragraphs 1-3, explains evolution of axillary management and need for study |
| Objectives | 3 | State specific objectives, including any prespecified hypotheses  ✓ YES - Introduction last paragraph, clearly states dual function of IO-USG |
| Methods | | |
| Study design | 4 | Present key elements of study design early in the paper  ✓ YES - Methods "Study Design" section, states "retrospective cohort study" |
| Setting | 5 | Describe the setting, locations, and relevant dates, including periods of recruitment, exposure, follow-up, and data collection  ✓ YES - Methods, single high-volume breast surgery clinic, January 2019-December 2023 |
| Participants | 6 | (a) Give the eligibility criteria, and the sources and methods of selection of participants. Describe methods of follow-up  ✓ YES - Methods "Patient Selection" section, clear inclusion/exclusion criteria |
|  |  | (b) For matched studies, give matching criteria and number of exposed and unexposed  NA - Not applicable for cohort study |
| Variables | 7 | Clearly define all outcomes, exposures, predictors, potential confounders, and effect modifiers. Give diagnostic criteria, if applicable  ✓ YES - Methods "Surgical Technique" section, IO-USG criteria defined (>3mm cortex, etc.) |
| Data sources/ measurement | 8* | For each variable of interest, give sources of data and details of methods of assessment (measurement). Describe comparability of assessment methods if there is more than one group  ✓ YES - Methods, retrospective chart review, frozen section analysis described |
| Bias | 9 | Describe any efforts to address potential sources of bias  ✓ YES - Discussion "Limitations" paragraph, selection bias acknowledged |
| Study size | 10 | Explain how the study size was arrived at  ✓ YES - Results first paragraph, n=314 consecutive patients |
| Quantitative variables | 11 | Explain how quantitative variables were handled in the analyses. If applicable, describe which groupings were chosen and why  ✓ YES - Statistical Analysis section, continuous and categorical variables defined |
| Statistical methods | 12 | (a)Describe all statistical methods, including those used to control for confounding  ✓ YES - Statistical Analysis section, t-test, Mann-Whitney, chi-square, logistic regression |
|  |  | (*b*) Describe any methods used to examine subgroups and interactions  ✓ YES - Results, three groups analyzed (SLNB-only, TAD, ALND) |
|  |  | (*c*) Explain how missing data were addressed  NO - Not reported in manuscript |
|  |  | (*d*) If applicable, explain how loss to follow-up was addressed  ✓ YES - Patient Selection, "incomplete follow-up data" excluded |
|  |  | (*e*) Describe any sensitivity analyses  NA - Not performed |
| Results | | |
| Participants | 13* | (a) Report numbers of individuals at each stage of study—eg numbers potentially eligible, examined for eligibility, confirmed eligible, included in the study, completing follow-up, and analysed  ✓ YES - Figure 2 flow diagram shows patient distribution |
|  |  | (b) Give reasons for non-participation at each stage  NO - Not reported |
|  |  | (c) Consider use of a flow diagram  ✓ YES - Figure 2 provides flow diagram |
| Descriptive data | 14* | (a) Give characteristics of study participants (eg demographic, clinical, social) and information on exposures and potential confounders  ✓ YES - Table 1, baseline characteristics (n=314) |
|  |  | (b) Indicate number of participants with missing data for each variable of interest  NO - Not reported |
|  |  | (c) Summarise follow-up time (eg, average and total amount)  ✓ YES - Results, median follow-up 33.2 months |
| Outcome data | 15* | Report numbers of outcome events or summary measures over time  ✓ YES - Results, 92/314 (29.3%) node-positive, detailed outcomes reported |
| Main results | 16 | (*a*) Give unadjusted estimates and, if applicable, confounder-adjusted estimates and their precision (eg, 95% confidence interval). Make clear which confounders were adjusted for and why they were included  ✓ YES - Table 3, OR with 95% CI for predictors |
|  |  | (*b*) Report category boundaries when continuous variables were categorized  NA - Categories not used |
|  |  | (*c*) If relevant, consider translating estimates of relative risk into absolute risk for a meaningful time period  NO - Relative risks reported, not absolute |
| Other analyses | 17 | Report other analyses done—eg analyses of subgroups and interactions, and sensitivity analyses  ✓ YES - Figure 3, Kaplan-Meier survival analysis (OS, DRFS, IDFS) |
| Discussion | | |
| Key results | 18 | Summarise key results with reference to study objectives  ✓ YES - Discussion first paragraph, 76.4% avoided ALND, 1.8% false-negative |
| Limitations | 19 | Discuss limitations of the study, taking into account sources of potential bias or imprecision. Discuss both direction and magnitude of any potential bias  ✓ YES - Discussion paragraph 7, single-center, retrospective design, selection bias |
| Interpretation | 20 | Give a cautious overall interpretation of results considering objectives, limitations, multiplicity of analyses, results from similar studies, and other relevant evidence  ✓ YES - Discussion, findings contextualized with Z0011, AMAROS, SOUND trials |
| Generalisability | 21 | Discuss the generalisability (external validity) of the study results  ✓ YES - Discussion, single-surgeon experience limits generalizability noted |
| Other information | | |
| Funding | 22 | Give the source of funding and the role of the funders for the present study and, if applicable, for the original study on which the present article is based  ✓ YES - Declarations section, "Funding: None |

*Give information separately for exposed and unexposed groups.

**Note:** An Explanation and Elaboration article discusses each checklist item and gives methodological background and published examples of transparent reporting. The STROBE checklist is best used in conjunction with this article (freely available on the Web sites of PLoS Medicine at http://www.plosmedicine.org/, Annals of Internal Medicine at http://www.annals.org/, and Epidemiology at http://www.epidem.com/). Information on the STROBE Initiative is available at http://www.strobe-statement.org.
